# Social Media Use Among Parents and Caregivers of Children With Rare Genetic Diseases: Scoping Review

**DOI:** 10.2196/77087

**Published:** 2025-11-28

**Authors:** Charlotte Davis, Laura Bogaert, John Powell, Karen Low

**Affiliations:** 1 Department of Population Health Sciences Bristol Medical School University of Bristol Bristol, null United Kingdom; 2 Nuffield Department of Primary Care Health Sciences University of Oxford Oxford United Kingdom

**Keywords:** rare disease, genetic disease, social media, online support group, parent

## Abstract

**Background:**

Caring for children with rare genetic disorders is challenging due to complex medical needs and limited information. Often, information is scarce due to geographical dispersion and lack of access to expertise. Social media groups are increasingly used in parenting and in healthcare as tools for data sharing and acquisition, and online peer support. Online groups relating to specific rare diseases are increasingly used by parents navigating the difficulties of understanding their child’s diagnosis and providing them with support. Parents expect professionals to interact with them about information reported from online groups, but little is known regarding the content within these groups and the impact on families.

**Objective:**

We aimed to synthesize current knowledge of social media use among parents and caregivers of children with a rare genetic syndrome to inform how these data might be used in parent-doctor interactions and in the research setting.

**Methods:**

We completed a comprehensive literature review across Web of Science, PubMed, and PsycINFO using a search strategy with themes of caregivers, rare genetic disease, and social media. Studies published in English from 2005 onwards, with parents and caregivers as a cohort and a focus on rare genetic diseases, were included. In total, 159 articles were identified, which underwent a title sift followed by an abstract sift based on inclusion and exclusion criteria. Reference lists of included articles were also reviewed. A total of 12 studies were included, and a critical synthesis methodology was used to extract relevant points.

**Results:**

Most parents and caregivers use social media platforms, especially Facebook (Meta Platforms, Inc), particularly the group function. They are using social media groups as a tool for finding information related to their child’s rare genetic disease. A majority also engaged in online groups by sharing information and contributions of their own. This review highlights that caregivers are seeking three main types of support from social media: (1) medical information around diagnosis and treatments, (2) practical tips on care needs and equipment, and (3) social support, involving connection with other families who shared similar experiences. The use of social media improved accessibility to information regardless of time or geography and reduced feelings of isolation. Caregivers felt empowered in decision-making, and their interactions with health care professionals improved. Challenges include misinformation, concerns around privacy, emotional impacts of comparison, and a lack of online spaces for the rarest conditions.

**Conclusions:**

Social media is a key tool for caregivers of children with rare genetic diseases. Addressing the associated challenges and harnessing the potential of these platforms can positively impact these families. Health care providers should consider discussing social media engagement in conversations with caregivers, and future research should focus on larger, longitudinal studies to explore the impacts of social media engagement.

## Introduction

### Rare Genetic Disease

Rare diseases are defined as those that affect one in 2000 people (European Commission); however, as a group, they are relatively common [1]. The UK Rare Diseases Framework states that 1 in 17 people in the United Kingdom are affected and that around 80% of these rare diseases are genetic, caused by a mutation within the DNA [2]. These mutations lead to altered function of proteins in the body, often resulting in widespread and multisystem impacts, such as developmental delay, congenital malformations, disorders of metabolism, and many more [3]. Most people are affected from childhood (75% of rare diseases) with a chronic, lifelong course, sometimes resulting in early death [2].

On top of the challenges of caring for a child with any health condition, parents and caregivers of children with rare genetic diseases face additional hurdles. The low prevalence of these conditions means there is often a lack of knowledge and scarcity of expertise among health care professionals [4,5]. Diagnosis can often be delayed or uncertain, and health care professionals cannot always meet the information needs of parents, particularly when diseases are newly described [6]. Potential support groups are geographically dispersed and very small, with limited resources available [7]. Parents need to find ways to gain knowledge of their child’s condition and develop caregiving skills to meet the specific needs of their child.

### Social Media Use

The advent of social media has revolutionized the way individuals seek and share information, particularly in health care contexts [8]. Parents use social media platforms, such as Facebook (Meta Platforms, Inc), Instagram (Meta Platforms, Inc), and YouTube (Google LLC) as a key resource for their children’s health information. The convenience of meeting and discussing health questions and issues with like-minded parents has made social media a central place for contemporary parenting [9].

Traditionally, health care providers were one of the few sources of health information. Now, parents can actively seek out alternative information via social media, which has an impact on their relationship with health care professionals [10].

Studies looking at the impact of social media on the relationship between patients and health care providers have shown that social media empowers patients to be more informed and involved in their own care, allowing for improved communication with medical professionals [11,12]. Health promotion on social media also encouraged patients to seek medical attention more willingly [11]. Most patients discuss information they have found online with their health care provider; however, physicians often react negatively to discussions involving information sourced from social media [11-13]. Social media use is also related to shorter relationships with health care providers, as online discussions prompted patients to seek specific providers [11].

Many parents may never physically meet other parents caring for a child with the same or similar genetic condition [5]. In this context, online support networks offer unique benefits. Connecting with people outside the family who share similar medical experiences or symptoms can provide emotional support and practical advice, which are often invaluable for families navigating the complexities of rare diseases [14]. Parents have even reported that online connections with other families provide more practical, specific, and applicable information than that from health care providers [15,16]. Studies also suggest that the use of genetic condition–specific Facebook groups and social media is more widespread than previously characterized [15].

Social media provides a more accessible platform for information sharing than other rare disease forums. In-person support groups are often challenging due to geographical dispersion and time constraints, which are especially relevant in rare disease communities [17]. Online support groups also provide a level of anonymity and reduced stigma that make it more likely for people to share personal information [17]. Other online communities, such as blogs or forums, may be more difficult to locate and engage with than social media groups. As the majority of people already use social media in their daily lives, they may find these sites easier to navigate.

### Aims and Objectives

A majority of rare diseases present during childhood, and therefore parents and caregivers are often the ones involved at the start of the journey, through the diagnostic process, and initial understanding of the condition [2]. Parents are required to take on a more practical care role, which presents a specific set of challenges and questions. Anecdotally, we see that parents are increasingly reporting participation in social media groups specific to their child’s rare condition. However, there is a lack of data regarding how parents interact with these groups, the content of the data shared in the groups, the impact of participation on the families, and the impact on parent-doctor interactions and relationships. This data could better inform professionals and researchers on the attitudes and dynamics of parental social media use and allow the development of ways to use social media to distribute educational resources around diagnostics, symptoms, equipment, and care needs.

Studies specifically exploring parents’ experiences with social media support groups within a rare disease context are scarce [16]. To our knowledge, there is no existing literature review that synthesizes the current evidence on this topic.

We focused on parents as a study cohort because they take on a different role than patients, with a specific set of informational needs. The needs of a parent are likely to differ from an adult patient who may have lived with their condition for many years.

Understanding how parents use social media and the type of information that they are seeking provides an insight into gaps in the current provision by health care professionals and services. We know that social media can be a useful tool for information sharing and social support networks [13]. By examining where parents’ informational and support needs are, this review can guide future development of ways to use social media to distribute educational resources around diagnostics, symptoms, equipment, and care needs. We can begin to explore whether clinician promotion of, or involvement in, these online landscapes could be beneficial, and how online groups might be used to expand our datasets and scope of future research in rare diseases. This review aims to map the existing evidence of how parents and other caregivers use social media. It explores the prevalence, dynamics, and potential outcomes of social media use among parents and caregivers of children diagnosed with a rare genetic syndrome.

## Methods

### Research Questions

A precursory literature review informed discussions between a clinical geneticist, a psychology researcher, and a clinician researcher with experience in digital health care. These discussions, considering key areas of importance, along with patient and public input (parents of children with genetic disorders and a member of a charity), led to the development of the research questions. No protocol exists for this study, as it is a scoping review. The PRISMA-ScR (Preferred Reporting Items for Systematic Reviews and Meta-Analyses extension for Scoping Reviews) used for structure can be found in Multimedia Appendix 1. The research questions are as follows:

1. In what ways do parents and caregivers of children with rare genetic syndromes use social media to understand and manage their child’s rare genetic syndrome?
2. When engaging with social media, what types of information and support are they looking for?
3. How does the use of social media impact the health and social outcomes of the parents and caregivers?

### Inclusion and Exclusion Criteria

Studies were included or excluded based on the criteria listed in Textbox 1.

**Inclusion and exclusion criteria.**

**Inclusion criteria**

- A focus on the dynamics of social media use for understanding and managing rare genetic diseases or syndromes
- Parents or caregivers of patients as a study population
- Published in English from 2005 onwards

**Exclusion criteria**

- Published before 2005 or published in languages other than English
- Patients or health care workers as a study population
- Addressed more common genetic syndromes (>1 in 2000 cases)

### Search Strategy

We searched Web of Science, PubMed, and PsycINFO databases to identify relevant literature that focused on social media use by parents and caregivers of patients with rare genetic diseases (<1 in 2000 as per the European Commission) [1]. The authors looked for additional articles by scanning the reference lists of included articles.

Given the limited research on this topic, we aimed to maximize the sensitivity of the search strategy by using keyword searching. Search terms were under 3 key domains: parent, caregiver, and family; genetic disease, rare disorder, and undiagnosed condition; and social media, internet use, and social networking. Synonyms across all 3 domains, including names of known rare genetic syndromes and key social media sites, were incorporated to create an initial search strategy.

The search was first applied to the Web of Science database in April 2024 to inform an earlier iteration of this work. We then repeated the search on December 30, 2024, across PubMed, Web of Science, and PsycINFO databases. An additional wider search term of “genetic neurodevelopmental disorder” was also added (after discussion) to improve the scope of the search. The search syntax entered into the Web of Science database is as follows:

(TS=(parent* OR mother* OR father* OR caregiver* OR caretaker* OR family OR families)) AND (TS=(“genetic disease*” OR “genetic disorder*” OR Rare NEAR/3 (disorder* OR disease* OR syndrome*) OR “Marfan Syndrome” OR “Prader-Willi Syndrome” OR “Angelman Syndrome” OR “Ehlers-Danlos Syndrome” OR “Williams Syndrome” OR “undiagnosed condition” OR “clinical genetic testing” OR “special health care needs” OR “CSHCN” OR “genetic neurodevelopmental disorder*”)) AND (TS=(“social media” OR ((Twitter OR Facebook OR YouTube OR LinkedIn) AND “support group*”) OR “social networking” OR Twitter OR Facebook OR Instagram OR YouTube OR LinkedIn OR “internet use” OR “internet usage” OR “online social support”))

The full search strategy can be found in Multimedia Appendix 2.

### Study Selection

After removing duplicates, database searches identified 159 references to which the eligibility criteria were applied during a title sift, followed by an abstract sift. This approach was taken as the criteria were specific enough to exclude many studies by the title alone. The title sift was undertaken by 2 authors, and studies were grouped into “include,” “exclude,” or “uncertain.” Studies coded as “include” or “uncertain” were then reviewed in the abstract sifts. The most common reasons for exclusion at the abstract sift were a population group other than parents or caregivers, and no specific focus on social media use.

Two authors independently completed searches and subsequent study selection processes, with CD only reviewing the results of the LB’s earlier search after completing their study selection.

Around 11 articles [14-16,18-25] were identified, 10 [14-16,18-21,23-25] of which had been included by both authors after the title and abstract sift, and 1 article [22], which was excluded by LB in the initial search but included by CD. This was resolved through discussion and was subsequently agreed upon for inclusion.

A review of the reference lists of included articles by both authors identified 1 additional article [26], leading to a total of 12 included articles [14-16,18-26]. The data were charted for the included studies under headings of study objectives, methodology, and key findings. The data were extracted by 2 authors and then combined into a spreadsheet.

### Ethical Considerations

Consent to participate and ethical approval were not required for this review as no human participants were involved.

## Results

### Evaluation

**Figure 1:**
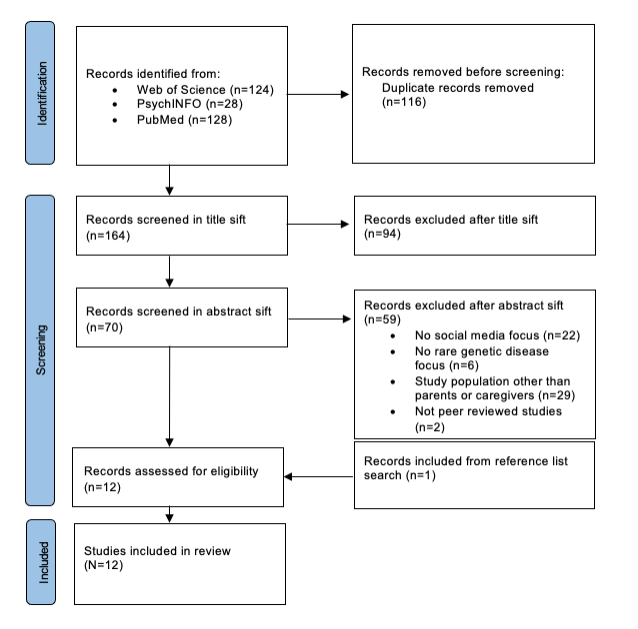
PRISMA (Preferred Reporting Items for Systematic Reviews and Meta-Analyses) diagram.

Figure 1 shows a PRISMA (Preferred Reporting Items for Systematic Reviews and Meta-Analyses) diagram to illustrate the study selection process. Completing 2 independent searches and study screening processes by independent authors with the same outcomes ensures low risk of bias in the study selection and demonstrates reproducibility.

A list of the 12 included studies [14-16,18-26] can be found in Table 1. The Mixed Methods Appraisal Tool was used to complete a bias assessment of included studies [27].

**Table 1:** A summary of characteristics for included articles.

Author and year	Objective	Methodology	Results
Barton et al [15], 2019	Analyze the use of both the internet and social media to help meet parents' informational and emotional support needs.	Qualitative method: nonstructured interviews of 20 parents whose children underwent clinical genetic testing	Parents use the internet to search for information at 3 stages of the genetic testing process: before testing, pending results return, and after results return. They use Facebook groups to learn more about their child’s condition and to find support networks, which would be challenging using nonsocial media approaches.
Cacioppo et al [16], 2016	Explore the experiences with internet support groups (ISGs) for parents of children with Cornelia de Lange syndrome (CdLS) and understand the impacts.	Mixed methods: 3 semistructured focus groups informing the design of an internet survey with 181 respondents	Around 141/170 respondents have visited ISG to find support or information about their child’s diagnosis. The majority of respondents reported that ISGs have been helpful in finding emotional and medical information support (behavior toward their children and family dynamic as the most important change).
Deuitch et al [14], 2021	Characterize how parents use social media, both throughout the diagnostic odyssey and post diagnosis, to meet their informational, social, and emotional support needs.	Qualitative semistructured interviews of 14 parents from the Stanford site of the Undiagnosed Diseases Network	Parents struggled to find the “right” community, seeking out groups of similar patients based on symptoms, and struggled to interpret the relevance of information to their child’s condition. Social support and access to others’ lived experiences are both highly valued and emotionally challenging, particularly when outcomes are poor. Parents need to balance concerns about their child’s privacy with the value of transparency and data sharing for diagnosis.
Iyer et al [24], 2020	Understand how patients and families with spinal muscular atrophy used social media to share, consume, and evaluate information about the novel treatment nusinersen following the drug’s approval.	Qualitative, semistructured interviews of 20 spinal muscular atrophy parents and patients	Participants leverage social media to learn about nusinersen treatment, make informed treatment decisions, and advocate for or access treatment. They described critically evaluating the trustworthiness of nusinersen-related information on social media and the privacy risks of social media use.
Nicholl et al [19], 2017	Ascertain parents’ general internet usage patterns, identify the nature of the information parents most frequently searched for, and determine the effect of the internet-sourced information.	Mixed methods: focus group (8 participants) to inform the questionnaire, which then had 128 responses	Parents frequently use the internet and social media to gather information on their child’s condition. Respondents reported that these resources positively impacted their decision-making, care, and management of their child. They reported receiving mixed responses when engaging their health care professionals about their social media interactions and information outcomes.
Titgemeyer and Schaaf [20], 2020	Provide a comprehensive quantitative analysis of the extent of Facebook usage for rare pediatric disease support groups and explore factors influencing disease representation on Facebook.	Mixed method: academic research with statistical analysis of Facebook disease- and group-describing parameters	Notably, 6398 Facebook support groups, representing 826 diseases, were found, and 69% were private groups. Group type, size, and activity varied between groups. Number of groups per disease increased with higher prevalence.
Titgemeyer and Schaaf [18], 2022	Map the opportunities Facebook offers as a tool for pediatric rare disease support groups by investigating its use, advantages, and limitations, including privacy concerns.	Cross-sectional online survey with 231 respondents	Among the participants, 59.7% reported a self-initiated search for the Facebook group, 24.2% received recommendations from their health professionals, and 12.6% received recommendations from someone else. In addition, 79.2% of participants agreed to have health professionals as members of their Facebook group. Group members expressed more concern about privacy issues in general than in their respective Facebook groups.
Wang and Lund [25], 2020	Examine the types of information that need to be expressed in an online Facebook group.	Qualitative content analysis approach	In total, 34 categories of information needs were identified, divided roughly into 5 groups—feeding, medical health care, pharmaceutical care, social activities, and community. Feeding and gastrointestinal, community, pharmacology, dermatology, and social barriers are the most frequent information needs.
Wittmeier et al [21], 2014	Conduct a descriptive and quantitative analysis of the use of a social media campaign for Hirschsprung disease.	Quantitative analysis of social media engagement	The most common question posted on the Facebook group is related to treatment for extreme diaper rash. Responsiveness assessment demonstrated that a question could receive 143 views and 20 responses within 2 hours, increasing to 30 responses after 5 hours.
Tozzi et al [23], 2013	Describe the Internet user profile of Italian families of patients with rare diseases, and explore how Internet use affects their health decisions.	Survey-based quantitative research with 516 survey respondents	Around 87% of respondents accessed the internet daily, and 71% had a Facebook account. Among the respondents, 99% searched for information on disease characteristics, 93% on therapy, 89% on diagnosis, 63% on alternative therapies, 62% on nutrition, and 54% on future pregnancies. People participating more frequently stated that Internet information was useful for recognizing their child’s disease and for improving its management.
Glenn [26], 2015	Explore the lived experience of using online health communications to manage chronic sorrow.	Phenomenological study with semistructured interviews of 16 mothers of children with Alagille syndrome	Analysis yielded 4 essential themes, such as connectedness, online triggers, empowerment, and seasons of online use, which contributed to online communication essential to a rare disease community.
Castro et al [22], 2019	Explore views of Osteogenesis Imperfecta (OI) caregivers on the use of internet-based technologies (IBTs) to support them in caring for their child with OI.	Qualitative method: semistructured interviews of 18 caregivers who brought a patient to an OI-related appointment	Overall, 14 of the 18 participants responded that they had a generally positive view of using IBTs in relation to caring for their child with OI. Caregivers, primarily mothers, were strategic in how they used OI social media. Most preferred social media for meeting other OI families. They shared day-to-day care information rather than using it for specific information on prognoses or treatments. One caregiver watched inspirational YouTube videos posted by families with OI to help motivate her daughter and herself.

All studies included clear aims and objectives, and study designs were appropriate to answer these. Where mixed methods were used, the rationale was clear. Participant numbers ranged from 14 to 516 respondents. It was noted that demographic data often showed a higher proportion of college-educated and married parents, which may indicate an element of nonresponse bias, with those with higher electronic literacy being more able to engage in the studies. All studies appropriately used either thematic analysis or descriptive statistics to analyze their results.

**Figure 2:**
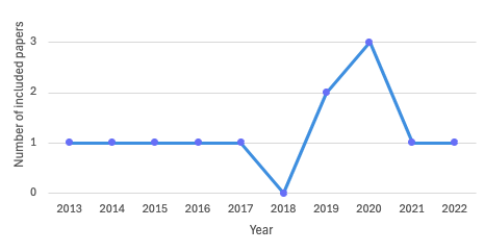
Line graph to represent the distribution of publication years of included studies.

Most studies used qualitative or mixed methods research methodology, and 9 studies [14-16,18,19,22-24,26] collected data using surveys or semistructured interviews to gain an understanding of parental views, mostly advertised through rare disease organizations. Around 5 [14-16,24,26] study cohorts were based in the United States, 1 [22] in Canada, and 2 [19,23] in Europe. Altogether, 4 studies [18,20,21,25] (2 US-based [21,25] and 2 Germany-based [18,20]) collected data using Facebook searches or groups that could include members across multiple countries. Advertisements on Facebook or other social media sites also increased the risk of sampling bias, as participants were more likely to already positively engage with social media. Figure 2 shows the distribution of publication years of included studies.

One study was identified as being at higher risk of bias, as there was no clear explanation for how the recruitment platforms were selected [18]. Recruitment via Facebook groups also introduced a high risk of sampling bias, as participants who engaged may be less likely to have concerns regarding limitations and privacy of the groups, which was a key research question. Table 1 summarizes the characteristics and findings of all included studies.

### Caregiver Usage of Social Media

***Engagemen***t

Most studies found that between 80% and 100% of parents are engaging in online support groups [14,16,19,20]. Facebook was the most commonly used platform and was found to have the largest proportion of support groups compared to other sites [14,16,21]. A significant reason for Facebook’s popularity was the group function, allowing communities to create online spaces to discuss and share information [19,22]. This feature is lacking on other social media platforms. One study found that at least 1 Facebook support group was identified for 19.5% of known rare genetic diseases, with 6398 groups identified in total, representing 826 diseases [20].

#### Information Sharing

Not only are parents accessing information via these groups, but also 4 studies [18,21,22,24] commented on the sharing of information. Of those caregivers who were engaging in social media support groups, 3 studies [16,19,23] reported that a majority (58.5%, 86%, and 81%, respectively) also actively contributed and shared information within the groups. Almost half of the included studies reported that parents were sharing their child’s personal experiences and stories within these groups [14,18,20,21,24]. Overall, 3 studies [14,24,25] noted that group members were sharing information on treatments, including medication, procedures, novel treatments, and alternative therapies. Altogether, 3 studies [20,21,24] highlighted that disease advocacy was often prevalent, with 1 study [20] reporting that 1 out of 10 online support groups identified were specifically targeted at raising awareness. Day-to-day care information and guidance were often shared, such as the practicalities of medication administration [22,24].

#### Need Dependent Variations in Social Media Use

Parents’ engagement in social media groups varied by circumstances and needs. Three studies [15,24,26] mentioned the increased use of online communities in stressful periods, such as initial diagnosis, receipt of results, and medical procedures.

Different platforms were used in different ways. Facebook was reported to provide guidance and experience from other families, personal story sharing, and information on events [21,24,26]. One study [23] noted that older parents were more familiar with Facebook and were less likely to use other sites. YouTube, as mentioned in 3 studies [16,22,26], was associated mainly with personal story sharing, with 1 study [22] reporting that parents find YouTube videos hopeful and inspirational. One study [14] reported that Instagram was used by over half of parents, but was also used for personal story-sharing accounts rather than discussion forums. Notably, 2 studies [19,21] mentioned the use of Twitter (subsequently rebranded X; X Corp), often used for similar content to Facebook posts but with less engagement. More traditional forums, such as blogs and advocacy groups, had negative connotations. One study [26] reported that parents feel blogs are associated with negative information about children in poor health. Another study reported that advocacy groups cannot offer the same responsiveness, interactivity, and community as Facebook groups [24].

Despite the extensive presence of support groups, parents of children with the rarest genetic diseases may find difficulties in accessing support. One study [20] highlighted that the odds of finding a Facebook support group for a disease with unknown prevalence are 4 times lower, with 326 people creating a group for a specific disease, but no one else joined. In such situations, parents may join several, more symptom-specific groups, but they sometimes feel like outsiders [14].

Two studies [14,20] that reviewed parental privacy concerns showed that parents are more comfortable joining and posting in private groups, and 60% of them were uncomfortable sharing in public groups, with private groups having triple the members. However, 1 study [14] highlighted that sometimes the urgent need for information can override these privacy concerns.

### Types of Support Seeking

***Overview***

Studies show that parents are primarily seeking medical information, practical advice, and community support [20,25].

#### Medical Information Finding

Eight studies [16,20-26] showed parental use of social media for medical information finding. This included searching for information on diagnoses, treatment options, specialist physicians, alternative therapies, and future pregnancies [23]. An example of this is shown in 1 study [24] where parents of children with spinal muscular atrophy used social media to gather information on nusinersen (a possible drug treatment) following its approval [24].

Four studies [14,16,23,26] also highlighted that the rarity of some diseases means providers cannot offer adequate information on the diagnosis, and therefore, parents rely more heavily on online communication for information. On the other hand, 2 studies [14,22] found parents prefer to receive medical and prognostic information from clinicians.

#### Practical Advice

Practical information on caring for a child with a rare genetic disease was sought or shared by parents in 9 [14-16,18,21-25] of the included studies. Parents found online communities have unique solutions based on personal experience, of which the key themes are summarized in Table 2. Some caregivers were more interested in answering others’ questions than having their own answered [22].

**Table 2:** Key categories of practical information sought and shared within online support communities.

Practical information themes	Specific practical topics by study
Access to providers	Specialist centers or clinicians [16,23] Second opinion [23] Centers offering novel treatment [24] Strategies to obtain services [15]
Insurance	Health, disability, and life insurance [16] Insurance challenges [24]
Care needs	Day-to-day care [16,18,21,22] Bowel management and potty training [21] Behavioral challenges [25] Fracture splinting [22] Seizures [14]
Equipment	Specialist equipment [14] Wheelchair adjustments, adaptive clothing, and specialist footwear [22]
Feeding	Feeding tubes [14] Diet [16,21] Feeding [25] Nutrition [23]
Development	Milestones, cognitive development [25] Immunization [23]
Physical Therapy	Therapy interventions [16] Movement [23] Physical activity [23]
Social	School system [16] Social barriers [16,25]

#### Community Support

Another main reason for joining online support networks was emotional support seeking and connection with other families, as found in 7 [14-16,19,20,23,26] of the reviewed studies. Caring for a child with a chronic illness can lead to distress and unpleasant feelings in caregivers [26]. Personal online communication with other families going through similar experiences helped parents to manage these emotions [15,20,26]. One study [18] reported that improved caregiver knowledge as a result of engagement in social media groups led to reduced stress. Personal experiences shared on social media with positive outcomes also provided parents with hope for their own child in the future [14,18,22]. However, several studies also noted that engagement with social media and online groups could trigger negative emotions and increase parental anxiety due to information overload and witnessing negative outcomes for other children [14,16,19,22,23,26]. One study [14] stated that parents recognized the emotional toll of social media and are careful about what they share to prevent troubling other families.

### Impact of Social Media Use on Caregiver Health and Social Outcomes

#### Reporting of Impacts

All studies included reported that parents were able to access information on social media, which enhanced their understanding and management of their child’s condition. Three studies [16,19,23] asked caregivers specific questions relating to the impact of their social media engagement. Further, 2 studies [22,26] used open questions, but with some inferred relation to outcomes. The remaining studies did not mention the impact on outcomes in the data collection process. None of the included studies had a longitudinal component, making it more difficult to ascertain clear cause-and-effect relationships.

##### Interactions With Health Care Providers

Parental discussion of online findings with health care providers was reported by 5 studies [15,19,23,24,26]. The online findings facilitated more informed and focused conversations and allowed parents to feel more valued and heard [15,26]. Moreover, 1 study [20] showed there are instances where online information led parents to question or even change health care providers. One study [14] noted that most of their respondents did not discuss social media use with health care professionals due to the negative stigma associated with using social media for information. Three studies [15,20,26] reported that some providers discouraged social media use or were uninterested in discussing the topic with caregivers. Two studies [18,21] noted that parents felt the presence of a health care provider in social media groups could reduce misinformation.

Three studies [14,15,22] brought focus to the accessibility of information via social media, with parents able to access it wherever and whenever they need it and receive faster answers to questions online than if they awaited input from health care providers. One study [15] also found that parents found more success in information seeking on social media compared to a simple internet search.

##### Social and Emotional Impacts

One study [16] asked specifically about the psychological impact of internet support groups and found that emotional support, solidarity, and understanding provided by social media groups positively influenced parental behavior toward the child affected and their siblings.

**Figure 3:**
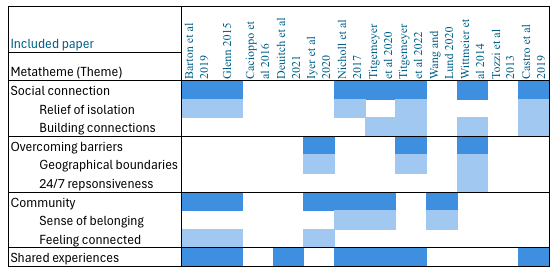
Thematic matrix showing themes and meta-themes of social support provided to parents and caregivers by social media and online support groups [15,25,16,14, 26,20,21,19,27,22,24,23].

Social isolation is common among parents of children with rare genetic diseases, especially when they have not yet received a diagnosis, as highlighted by 3 studies [14,15,26]. Most of the included articles commented on the social support provided by social media, the key themes of which have been represented in Figure 3. One study [26] highlighted that although online social support had many benefits, parents recognized that it could be challenging to support others and that online support could not completely replace in-person interactions. Parents whose children were undiagnosed or had very rare diseases often struggled to find the right groups to connect with, and so feelings of isolation remained [14].

## Discussion

### Social Media Outcomes

This review revealed that a majority of parents and caregivers are using social media and primarily through group functions, especially on Facebook. Within these groups, they are both seeking and sharing information about their child’s rare genetic condition. We identified 3 main themes regarding the content of the information sought and shared: (1) medical information around diagnosis, treatments, and providers; (2) emotional support and connections with other families; and (3) practical tips, such as specialist equipment. Positive outcomes of engagement in social media include easier and faster access to information and building social support networks. However, some negative outcomes were also recognized, such as concerns around privacy, emotional impacts of comparison, and lack of online spaces for the rarest conditions.

### Principal Findings

***Overview***

The prevalence of social media use by parents and caregivers shown in this review is indicative of the increasing use of these platforms both in society and in health care. The rising number of social media support groups highlights a growing reliance on digital platforms for information sharing and emotional support, especially in communities where geographical dispersal and intense care needs of the child present barriers to in-person parental meetings or training [28]. Online platforms allow parents almost immediate access to information or support at any time of the day or night, and no matter their location.

#### Satisfaction of Informational Needs

This review shows that the categories of information sought and shared by caregivers on social media are broadly in line with each other. Specifically, caregiver needs for information on day-to-day care, access to services, and treatment options appear to be met by social media platforms, as well as their desire to connect with other individuals and families. Parents were seeking medical information on diagnosis and symptoms, and although personal experiences were often shared, it is unclear if reliable evidence-based information on this was available to them, with misinformation being a key risk of information searching online. Without the presence of medical professionals within groups, it may be difficult to validate information. Some studies report “fact-checking culture” within online groups, although the methods by which this occurs are not explored [24].

Social media groups also served as a powerful space for emotional and social support among parents, so much so that some developed offline relationships [22]. Some parents found hope and inspiration in the experiences of others; however, others reported negative impacts, and it may be useful to consider whether these parents had access to formal emotional or psychological support services. Extensive information on the content of social media posts was lacking from several studies, with some acknowledging that further research is needed in this area [15]. It is also difficult to ascertain how streamlined the pages are from the studies included in this review and whether parents and caregivers must sift through irrelevant or unhelpful information in order to find what they are seeking. This is not something covered by this review, but exploring the content, quality, and volume of posts shared in these social media groups could be a potential focus for future research.

The overall impact of online support groups was assessed by 2 studies [16,22], both of which reported that over half of respondents viewed them as positive. Most other studies did not report on this specifically but presented both positive and negative points relating to parent and caregiver use of social media. Two studies [16,18] reported that most participants visited online support groups at least once per day. Parents acknowledged the balance of consuming too much information that may not be relevant while ensuring that they stay up to date with posts to avoid missing important information [14]. None of the included studies commented on the duration of social media use or the effects of long-term use. Further research is required to understand the impacts of prolonged and frequent engagement in online support groups.

Regarding disease advocacy, social media plays a role in raising awareness and visibility of rare diseases in the hope of engaging a broader audience and subsequently improving funding, research, and specialist care opportunities. Improved parental knowledge and understanding of their child’s conditions facilitates well-informed decision-making and discussions with health care providers, which ultimately improves outcomes for the child. Lower levels of health literacy among parents of children with chronic disease are associated with poor health outcomes, more medical errors, lower treatment adherence, and increased hospitalization rates [29]. Without online platforms for information sharing, parents may find it difficult to access up-to-date information, often in the form of medical academic literature, due to jargon and paywalls [25].

#### Social Media Use by Other Groups Within Rare Disease

One study [30] was identified but excluded during the literature search, as it focused on social media usage in parents of children with FIRES (Febrile infection-related epilepsy syndrome), a rare disease that is not genetic in origin. The findings mirrored those of this review, with parents reporting Facebook groups to be beneficial, especially in emotional support. This demonstrates that social media groups are also used across the spectrum of rare diseases of all etiologies and that many of the learnings are applicable more widely, even to less rare disorders. Similarly, 3 excluded studies [31-33] focused on the use of social media support groups by patients with rare diseases. The ways in which parents and patients access information online are very similar, with online support groups serving as the most common mechanism and Facebook being the most common platform in both cohorts.

Patients with rare genetic diseases use social media to overcome the same boundaries that were found by parents in this review, including geographical dispersion, limited medical expertise, and connection with peers [34]. The information that patients are seeking falls into some of the same categories of information that parents and caregivers sought. Patients search for other people with similar conditions to connect with and want to feel part of a community [31-35]. They also often search for advice based on others’ experience, such as on novel treatments, medications, physicians, and nonmedical therapies [31,35]. Searching primarily for these topics of management strategies and social connection suggests that these patients already have a basis of knowledge, likely from their own experience of their condition. This is where social media use by parents and caregivers differs from patients, as parents were seeking information about diagnosis, symptoms, and more practical advice about care procedures and equipment, which may reflect their lack of experience with the disease. However, some rare diseases are diagnosed in adulthood, and diagnosis was highlighted as an initiating factor in seeking online support and information in these patients, similarly to the pattern seen in caregivers [33,34].

Privacy concerns and distrust of information online were significant concerns raised by patients regarding social media use for health information [34,36,37]. Comparatively, parents and caregivers did raise some concerns about privacy and misinformation, but these were much weaker themes than in the patient cohorts.

Patients of some rare genetic diseases may be affected by intellectual disability, which may make them more vulnerable online. A study focused on individuals with Williams syndrome showed that a majority of participants used social networking on a daily basis; however, they were more likely to engage in socially risky behaviors and share large amounts of information on their profiles [38]. Therefore, the ability of this cohort of patients to engage in online support groups, and the format in which they may do this, will be very different from parents and caregivers.

Two further studies [36,37], which were excluded from the review due to their focus on patients rather than parents, looked at adolescent and young adult patients with rare and chronic diseases. They found that only a minority of young adult patients discussed their health information on social media. Those who did use social media did so with similar intentions to older adult patients: to connect with and talk to others with similar health issues and to gain information on symptoms and treatments, especially in emergencies when in-person resources were unavailable [36,37]. Young adult patients had significant concerns regarding online privacy and misinformation [36,37].

Health care provider engagement in social media is something not covered by many studies in this review, likely due to the focus on parental use. However, 1 study [18] mentioned that health care providers do not routinely recommend online support to parents, stating that less than 25% were directed to Facebook groups by their clinicians compared to 60% who self-initiated searches for online groups.. Most parents and caregivers discussed information they had found online with their health care providers; however, some experienced negative responses from health care professionals [14,15,19,26].

### Implications for Practice

For parents and caregivers, this review highlights the benefits of engagement in the online community as a source of information that can be used alongside medical advice, as well as a space to gain social and emotional support from other parents and caregivers. They must be wary of the potential for misinformation and the negative emotional impact that sharing and consuming the experiences of others can have. Parents should be encouraged, signposted, and empowered to seek out information and understanding of their child’s condition.

The findings highlight a missed opportunity for health care providers to engage in social media communities in order to provide reliable information and appropriate resources. Health care providers’ involvement would mitigate the risk of misinformation. There are mixed opinions on whether physician involvement in these groups would be welcomed by parents and caregivers, although 2 studies [18,21] highlight that parents would like health care professionals as members. For physicians and some health care providers, professional organization policies explicitly discourage professionals from engaging in social media for health care advice delivery to members of the public [39]. Updating such guidance could enable health care professionals to feel confident in promoting research-backed information sharing on social media.

Health care professionals may be able to improve their own online communication with disease experts and research communities to improve provider education on rare genetic diseases, which in turn allows them to improve the care they provide to patients and caregivers. At least, health care providers should consider discussing social media use with patients and caregivers at consultations to allow a space for information clarification, directing them to reputable resources and communities. By accessing reliable educational resources, patients and caregivers can improve their understanding, allowing for more informed engagement in their own or their child’s care.

Social media designers should consider the use of group functions on their sites and whether, with the increasing prevalence of online support networks, group features should be introduced on a wider range of platforms. Also, rare disease advocacy groups and organizations should ensure that they are using social media groups as a platform for their own online discussion networks and advocacy campaigns. For the rarest genetic diseases, where it can be difficult for parents to find others with similar experiences, disease organization social media networks could support links between families across the globe who are affected by the same rare genetic conditions.

### Strengths and Limitations

This review included a comprehensive literature search across several relevant databases. Completing independent searches by 2 separate authors reduced the chance of bias and created a more replicable set of results. We identified clear and specific research questions that could be appropriately answered by the included studies, with consideration for risk of bias.

A substantial proportion of the qualitative studies included in this review had relatively limited sample sizes, often involving 15-20 participants. This small sample size, largely due to the rarity of diseases, might introduce bias and could limit the generalizability of findings. Many studies recruited participants through social media, which might introduce selection bias, as participants are those already engaged in online communities. Additionally, some studies are based on data from the early 2010s, when internet access and social media use were less prevalent [21,23,26]. Furthermore, most studies are conducted in English, focusing on English-speaking populations and potentially excluding significant portions of the global community. Despite this, however, most online support groups are international and are therefore likely to be representative of a widespread international population.

Moreover, most studies offer only a snapshot of social media use at a single time point, with no longitudinal data to consider the changes in experience over time as children age and parents gain understanding of their child’s condition. While many of the studies included in this review report perceived benefits to parental understanding and emotional well-being, this is often not measured using standardized variables. Considering the methodological process, the search terms could also have been expanded to include a broader list of rare genetic disease names, potentially capturing a more diverse set of insights, including ultrarare diseases.

### Recommendations for Future Work

We have identified that many parents use social media as a key source of information gathering and education; however, the quality of medical information on these platforms has not been explored. Future research could consider the impact of medical information and misinformation shared on social media on decision-making and child health outcomes. Studies could also look at interventions to improve parental digital literacy and the ability to critically evaluate online information. This could inform the development of caregiver education programs.

Investigating how health care professionals’ engagement and contributions to these platforms impact the quality of information, parental satisfaction, and health outcomes for children would also help develop an understanding of information dissemination strategies in the future. We know that many parents are using social media, and often as a source of emotional and social support; however, the long-term effects of this are little understood. Longitudinal studies considering the sustained benefits and risks associated with prolonged engagement in these communities are also needed. These would also be beneficial given the rapidly changing landscape of social media platforms and groups.

In considering future research limitations, one major obstacle is the difficulty of performing research within social media groups, when companies such as Meta do not allow access for researchers. Many groups, as discussed earlier, are closed for privacy concerns, making it difficult to access without aid from the group itself. Gaining consent to use data from online posts would be ethically difficult to obtain for longitudinal use, which is a concern given the ease of searching and accessing posts. Consent and reconsent issues would also apply to children who turn 16 years old. In the United Kingdom, for example, current National Health Service (NHS) policy also often restricts health care providers from using social media in order to protect patient confidentiality and maintain a clear boundary between personal and professional for healthcare providers.

### Conclusions

This literature review has synthesized available research on the use of social media by parents of children with rare genetic diseases. The widespread use of social media is evident, especially Facebook groups, in parental information seeking, experience sharing, practical tips, and emotional support. While these are valuable platforms, risks around misinformation and unnecessary anxiety can occur. Health care providers should consider how engagement in these online communities, either directly or in conversations with parents and caregivers, can support understanding and empowerment with the hope of improved patient care.

Future research should focus on larger, more diverse studies to explore the impacts of social media-sourced information, as well as longitudinal studies to consider the long-term patterns and implications of social media engagement. Social media is an increasingly accessed source of information for parents and caregivers of children with rare genetic diseases. By addressing challenges and leveraging the potential of these platforms, support systems, and outcomes for these families can be improved.

## Data Availability

Data sharing is not applicable to this article as no datasets were generated or analyzed during this study.

## Appendix

Multimedia Appendix 1 PRISMA-ScR checklist.

Multimedia Appendix 2 Full search strategy.

## Abbreviations

FIRES: Febrile infection-related epilepsy syndrome
NHS: National Health Service
PRISMA: Preferred Reporting Items for Systematic Reviews and Meta-Analyses
PRISMA-ScR: Preferred Reporting Items for Systematic Reviews and Meta-Analyses extension for Scoping Reviews

## References

[1] Rare diseases. European Commission.

[2] The UK Rare Diseases Framework. UK Department of Health and Social Care.

[3] Lee CE, Singleton KS, Wallin M, Faundez V (2020). Rare genetic diseases: nature's experiments on human development. iScience.

[4] Nguengang Wakap S, Lambert DM, Olry A, Rodwell C, Gueydan C, Lanneau V, Murphy D, Le Cam Y, Rath A (2020). Estimating cumulative point prevalence of rare diseases: analysis of the Orphanet database. Eur J Hum Genet.

[5] Pelentsov LJ, Fielder AL, Laws TA, Esterman AJ (2016). The supportive care needs of parents with a child with a rare disease: results of an online survey. BMC Fam Pract.

[6] Baumbusch J, Mayer S, Sloan-Yip I (2018). Alone in a crowd? Parents of children with rare diseases' experiences of navigating the healthcare system. J Genet Couns.

[7] Pelentsov LJ, Laws TA, Esterman AJ (2015). The supportive care needs of parents caring for a child with a rare disease: a scoping review. Disabil Health J.

[8] Frey E, Bonfiglioli C, Frawley J (2023). Parents' use of social media for health information before and after a consultation with health care professionals: Australian cross-sectional study. JMIR Pediatr Parent.

[9] Oprescu F, Campo S, Lowe J, Andsager J, Morcuende JA (2013). Online information exchanges for parents of children with a rare health condition: key findings from an online support community. J Med Internet Res.

[10] Breivold J, Rø KI, Hjörleifsson S (2022). Conditions for gatekeeping when GPs consider patient requests unreasonable: a focus group study. Fam Pract.

[11] Smailhodzic E, Hooijsma W, Boonstra A, Langley DJ (2016). Social media use in healthcare: a systematic review of effects on patients and on their relationship with healthcare professionals. BMC Health Serv Res.

[12] Benetoli A, Chen TF, Aslani P (2018). How patients' use of social media impacts their interactions with healthcare professionals. Patient Educ Couns.

[13] Forgie EME, Lai H, Cao B, Stroulia E, Greenshaw AJ, Goez H (2021). Social media and the transformation of the physician-patient relationship: Viewpoint. J Med Internet Res.

[14] Deuitch NT, Beckman E, Halley MC, Young JL, Reuter CM, Kohler J, Bernstein JA, Wheeler MT, Ormond KE, Tabor HK, Undiagnosed Diseases Network (2021). "Doctors can read about it, they can know about it, but they've never lived with it": how parents use social media throughout the diagnostic odyssey. J Genet Couns.

[15] Barton KS, Wingerson A, Barzilay JR, Tabor HK (2019). "Before Facebook and before social media…we did not know anybody else that had this": parent perspectives on internet and social media use during the pediatric clinical genetic testing process. J Community Genet.

[16] Cacioppo CN, Conway LJ, Mehta D, Krantz ID, Noon SE (2016). Attitudes about the use of internet support groups and the impact among parents of children with Cornelia de Lange syndrome. Am J Med Genet C Semin Med Genet.

[17] Strand M, Eng LS, Gammon D (2020). Combining online and offline peer support groups in community mental health care settings: a qualitative study of service users' experiences. Int J Ment Health Syst.

[18] Titgemeyer SC, Schaaf CP (2022). Facebook support groups for pediatric rare diseases: cross-sectional study to investigate opportunities, limitations, and privacy concerns. JMIR Pediatr Parent.

[19] Nicholl H, Tracey C, Begley T, King C, Lynch AM (2017). Internet use by parents of children with rare conditions: Findings from a study on parents' web information needs. J Med Internet Res.

[20] Titgemeyer SC, Schaaf CP (2020). Facebook support groups for rare pediatric diseases: quantitative analysis. JMIR Pediatr Parent.

[21] Wittmeier K, Holland C, Hobbs-Murison K, Crawford E, Beauchamp C, Milne B, Morris M, Keijzer R (2014). Analysis of a parent-initiated social media campaign for Hirschsprung's disease. J Med Internet Res.

[22] Castro AR, Chougui K, Bilodeau C, Tsimicalis A (2019). Exploring the views of osteogenesis imperfecta caregivers on internet-based technologies: qualitative descriptive study. J Med Internet Res.

[23] Tozzi AE, Mingarelli R, Agricola E, Gonfiantini M, Pandolfi E, Carloni E, Gesualdo F, Dallapiccola B (2013). The internet user profile of Italian families of patients with rare diseases: a web survey. Orphanet J Rare Dis.

[24] Iyer AA, Barzilay JR, Tabor HK (2020). Patient and family social media use surrounding a novel treatment for a rare genetic disease: a qualitative interview study. Genet Med.

[25] Wang T, Lund B (2020). Categories of information need expressed by parents of individuals with rare genetic disorders in a facebook community group: a case study with implications for information professionals. J Cons Hlth Internet.

[26] Glenn AD (2015). Using online health communication to manage chronic sorrow: mothers of children with rare diseases speak. J Pediatr Nurs.

[27] Hong QN, Fàbregues S, Bartlett G, Boardman F, Cargo M, Dagenais P, Gagnon M, Griffiths F, Nicolau B, O’Cathain A, Rousseau M, Vedel I, Pluye P (2018). Mixed Methods Appraisal Tool (MMAT), version 2018.

[28] DeHoff BA, Staten LK, Rodgers RC, Denne SC (2016). The role of online social support in supporting and educating parents of young children with special health care needs in the United States: a scoping review. J Med Internet Res.

[29] Tschamper MK, Larsen MH, Wahl AK, Jakobsen R (2023). Developing and maintaining health literacy: a continuous emotional, cognitive, and social process for parents of children with epilepsy-A qualitative study. Epilepsy Behav.

[30] Farias-Moeller R, Wood A, Sawdy R, Koop J, Olson K, van Baalen A (2021). Parental perception of FIRES outcomes, emotional states, and social media usage. Epilepsia Open.

[31] Ashtari S, Taylor AD (2022). The internet knows more than my physician: qualitative interview study of people with rare diseases and how they use online support groups. J Med Internet Res.

[32] Ashtari S, Taylor A (2023). Patients with rare diseases and the power of online support groups: implications for the medical community. JMIR Form Res.

[33] Rocha HM, Savatt JM, Riggs ER, Wagner JK, Faucett WA, Martin CL (2018). Incorporating social media into your support tool box: points to consider from genetics-based communities. J Genet Couns.

[34] Pearce EE, Majid A, Brown T, Shepherd RF, Rising C, Wilsnack C, Thompson AS, Gilkey MB, Ribisl KM, Lazard AJ, Han PK, Werner-Lin A, Hutson SP, Savage SA (2024). "Crying in the Wilderness"-The use of web-based support in telomere biology disorders: thematic analysis. JMIR Form Res.

[35] Doyle TA, Vershaw SL, Conboy E, Halverson CME (2024). Improving social media-based support groups for the rare disease community: interview study with patients and parents of children with rare and undiagnosed diseases. JMIR Hum Factors.

[36] Kelleher EF, Giampietro PF, Moreno MA (2020). Social media use among young adults with connective tissue disorders: cross-sectional pilot study. JMIR Pediatr Parent.

[37] Anikputa BC, Horner SD (2021). Internet use behavior among adolescents and young adults with chronic illnesses. J Pediatr Nurs.

[38] Lough E, Fisher MH (2016). Internet use and online safety in adults with Williams syndrome. J Intellect Disabil Res.

[39] Social media, ethics and professionalism BMA guidance. British Medical Association.

