# Supplementary material for: Social Media Use Among Parents and Caregivers of Children With Rare Genetic Diseases: Scoping Review
Source: J Med Internet Res. 2025 Nov 28;27:e77087. doi: 10.2196/77087 (PMC12701349; doi:10.2196/77087)
Supplement: Multimedia Appendix 2 [file jmir_v27i1e77087_app2.docx]

*Appendix A. Full Search Strategy*

| Ovid Psycho INFO .mp search [.mp=title, abstract, heading word, table of contents, key concepts, original title, tests & measures, mesh word] (31/12/2024) | |
| --- | --- |
| #1 | (parent* or mother* or father* or caregiver* or caretaker* or family or families).mp. |
| #2 | ("genetic disease*" or "genetic disorder*" or (Rare adj3 (disorder* or disease* or syndrome*)) or "Marfan Syndrome" or "Prader-Willi Syndrome" or "Angelman Syndrome" or "Ehlers-Danlos Syndrome" or "Williams Syndrome" or "undiagnosed condition" or "clinical genetic testing" or "special health care needs" or "CSHCN" or "genetic neurodevelopmental disorder*").mp. |
| #3 | ("social media" or ((Twitter or Facebook or YouTube or LinkedIn) and "support group*") or "social networking" or Twitter or Facebook or Instagram or YouTube or LinkedIn or "internet use" or "internet usage" or "online social support").mp. |
| #4 | #1 AND #2 AND #3 |
| #5 | Limit #4 to yr ‘2005’ to ‘2024’ |
| PubMed ‘all fields’ search (31/12/2024) | |
| #1 | (parent* OR mother* OR father* OR caregiver* OR caretaker* OR family OR families) |
| #2 | ("social media" OR ((Twitter OR Facebook OR YouTube OR LinkedIn) AND "support group*") OR "social networking" OR Twitter OR Facebook OR Instagram OR YouTube OR LinkedIn OR "internet use" OR "internet usage" OR "online social support") |
| #3 | ("genetic disease*" OR "genetic disorder*" OR (("rare disorder" [tiab:~3]) OR ("rare disorders" [tiab:~3]) OR ("rare disease" [tiab:~3]) OR ("rare diseases" [tiab:~3]) OR ("rare syndrome" [tiab:~3]) OR ("rare syndromes" [tiab:~3])) OR "Marfan Syndrome" OR "Prader-Willi Syndrome" OR "Angelman Syndrome" OR "Ehlers-Danlos Syndrome" OR "Williams Syndrome" OR "undiagnosed condition" OR "clinical genetic testing" OR "special health care needs" OR "CSHCN" OR "genetic neurodevelopmental disorder*") |
| #4 | (2005:2024[pdat]) |
| #5 | #1 AND #2 AND #3 AND #4 |
| #6 | Filter to #5 Language: English |
| Web of Science TS search [TS=Topic terms across Title, Abstract, Author Keywords, Keywords Plus^®^] (31/12/2024) | |
| #1 | TS=(parent* OR mother* OR father* OR caregiver* OR caretaker* OR family OR families) |
| #2 | TS=("genetic disease*" OR "genetic disorder*" OR Rare NEAR/3 (disorder* OR disease* OR syndrome*) OR "Marfan Syndrome" OR "Prader-Willi Syndrome" OR "Angelman Syndrome" OR "Ehlers-Danlos Syndrome" OR "Williams Syndrome" OR "undiagnosed condition" OR "clinical genetic testing" OR "special health care needs" OR "CSHCN" OR "genetic neurodevelopmental disorder*")) |
| #3 | TS= ("social media" OR ((Twitter OR Facebook OR YouTube OR LinkedIn) AND "support group*") OR "social networking" OR Twitter OR Facebook OR Instagram OR YouTube OR LinkedIn OR "internet use" OR "internet usage" OR "online social support")) |
| #4 | #1 AND #2 AND #3 |
| #5 | Filter #4 by date 2005-2024 |
